# Estimating 3‐D whole‐body composition from a chest CT scan

**DOI:** 10.1002/mp.15821

**Published:** 2022-07-11

**Authors:** Lucy Pu, Syed F. Ashraf, Naciye S. Gezer, Iclal Ocak, Daniel E. Dresser, Joseph K. Leader, Rajeev Dhupar

**Affiliations:** ^1^ Department of Cardiothoracic Surgery University of Pittsburgh School of Medicine Pittsburgh Pennsylvania USA; ^2^ North Allegheny Senior High School Wexford Pennsylvania USA; ^3^ Department of Radiology University of Pittsburgh School of Medicine Pittsburgh Pennsylvania USA; ^4^ Department of Pathology University of Pittsburgh School of Medicine Pittsburgh Pennsylvania USA; ^5^ Surgical Services Division VA Pittsburgh Healthcare System Pittsburgh Pennsylvania USA

**Keywords:** body composition, chest, computed tomography, prediction models, whole‐body

## Abstract

**Background:**

Estimating whole‐body composition from limited region‐computed tomography (CT) scans has many potential applications in clinical medicine; however, it is challenging.

**Purpose:**

To investigate if whole‐body composition based on several tissue types (visceral adipose tissue [VAT], subcutaneous adipose tissue [SAT], intermuscular adipose tissue [IMAT], skeletal muscle [SM], and bone) can be reliably estimated from a chest CT scan only.

**Methods:**

A cohort of 97 lung cancer subjects who underwent both chest CT scans and whole‐body positron emission tomography‐CT scans at our institution were collected. We used our in‐house software to automatically segment and quantify VAT, SAT, IMAT, SM, and bone on the CT images. The field‐of‐views of the chest CT scans and the whole‐body CT scans were standardized, namely, from vertebra T1 to L1 and from C1 to the bottom of the pelvis, respectively. Multivariate linear regression was used to develop the computer models for estimating the volumes of whole‐body tissues from chest CT scans. Subject demographics (e.g., gender and age) and lung volume were included in the modeling analysis. Ten‐fold cross‐validation was used to validate the performance of the prediction models. Mean absolute difference (MAD) and *R*‐squared (*R* ^2^) were used as the performance metrics to assess the model performance.

**Results:**

The *R* ^2^ values when estimating volumes of whole‐body SAT, VAT, IMAT, total fat, SM, and bone from the regular chest CT scans were 0.901, 0.929, 0.900, 0.933, 0.928, and 0.918, respectively. The corresponding MADs (percentage difference) were 1.44 ± 1.21 L (12.21% ± 11.70%), 0.63 ± 0.49 L (29.68% ± 61.99%), 0.12 ± 0.09 L (16.20% ± 18.42%), 1.65 ± 1.40 L (10.43% ± 10.79%), 0.71 ± 0.68 L (5.14% ± 4.75%), and 0.17 ± 0.15 L (4.32% ± 3.38%), respectively.

**Conclusion:**

Our algorithm shows promise in its ability to estimate whole‐body compositions from chest CT scans. Body composition measures based on chest CT scans are more accurate than those based on vertebra third lumbar.

## 1 INTRODUCTION

Body composition is strongly associated with an individual's health status and various clinical conditions, such as cancer,^1^ osteoporosis,^2^ cardiovascular diseases,^3^ and diabetes.^4^ Awareness of body composition can signal current and future health risks, facilitate individualized therapy, and monitor therapeutic performance. There are several ways to assess body composition, such as body mass index, waist circumference, bioimpedance, dual‐energy X‐ray absorptiometry (DXA), computed tomography (CT), and magnetic resonance imaging. Among these modalities, CT is gaining an increasing interest to quantitatively assess in vivo body composition because of its volumetric characteristics, high spatial and temporal resolutions, and wide use in clinical practice. In addition to bone and muscle, CT imaging can discriminate subcutaneous and visceral fat from each other and even visualize fat infiltration in skeletal muscle (SM) and liver. In clinical practice, a CT scan is acquired for a particular region of the body (e.g., chest or abdomen) as related to the disease. However, it would be desirable to have a clear concept of whole‐body composition because it may provide an assessment of an individual's overall health status and allow clinicians to better understand the implications of body composition on disease states.

Several studies have investigated if body composition in a single region of the body can sufficiently reflect an individual's overall body composition.^5^, ^6^, ^7^, ^8^, ^9^, ^10^, ^11^, ^12^, ^13^ The body composition obtained at the third lumbar (L3) vertebra was often used to estimate a subject's body composition.^6^, ^7^, ^8^ Swartz et al.^9^ reported that the cross‐sectional area of the SM at the third cervical (C3) vertebra strongly correlates with the cross‐sectional area of the SM at L3. Mishra et al.^10^ concluded that both L3 and the fourth thoracic (T4) vertebrae were useful locations for assessing body composition related to SM and adipose tissue. However, Gronberg et al.^11^ reported only a moderate agreement between the SM at the T4 and L3 and concluded that analyzing images at the T4 level could not replace the estimates of SM derived at L3. Most studies only investigated the correlation between the areas of body tissues from single images (e.g., at the level of C3, T4, or L3) or the total volumes of body tissues depicted on a chest or abdominal CT scan. Although body composition estimated on a single image or total volumes from a single region can reflect whole‐body composition, there are limitations with these techniques.^14^

In this study, we collected a cohort of 97 subjects with paired chest and whole‐body CT scans. The latter originated from positron emission tomography (PET)‐CT scans and typically covered the body regions from neck to thigh. Using our in‐house software, we quantified five different tissues related to body composition (visceral adipose tissue [VAT], subcutaneous adipose tissue [SAT], intermuscular adipose tissue [IMAT], SM, and bones). Additionally, lung volume was computed using the chest CT scans to study whether including this would improve estimation accuracy. Our objective was to develop and validate a set of computer models for estimating three‐dimensional (3‐D) whole‐body composition measures based on chest CT scans alone.

## 2 METHODS AND MATERIALS

### 2.1 Study cohort

To establish the cohort for this study, we revisited a previous study cohort consisting of 471 lung cancer subjects treated at our institution. The original cohort had the following inclusion criteria: (1) non‐small cell lung cancer, (2) pretreatment chest CT scans and PET‐CT scans, (3) posttreatment chest CT scans and PET‐CT scans, (4) lung resection, and (5) minimum 5‐year follow‐up. For this retrospective study, we applied additional inclusion criteria: (1) the pretreatment chest CT scans and the PET‐CT scans were acquired within 2 weeks of one another to ensure that their weight changes were limited; (2) the PET‐CT scans were acquired from head to thigh; and (3) the chest CT scans fully covered the body regions from T1 to L1. Ninety‐seven subjects met all inclusion criteria (Table 1). There were 45 men (46%) and 52 women (54%), with an average age of 67 (39–89). This study was approved by the University of Pittsburgh Institutional Review Board (IRB) (IRB #: STUDY20100305).

**TABLE 1:** Subject demographics in our study cohort

	All subjects (*n* = 97)	Male (*n* = 45)	Female (*n* = 52)
**Age, mean (range)**	67 (39–89)	68 (51–89)	66 (39–86)
**Race, *n* (%)**			
White	86 (89)	45 (100	41 (79)
African American	11 (11)	0 (0)	11 (21)
Other races	0 (0)	0 (0)	0 (0)
**Body weight (kg), mean (SD)**	76.72 ± 17.84	85.14 ± 16.16	69.44 ± 16.03
**Body mass index (kg/m^2^), mean (SD)**	27.0 ± 5.4	27.9 ± 5.2	26.2 ± 5.6
Underweight, ≤18.5, *n* (%)	3 (3)	0 (0)	3 (6)
Normal weight, 18.5–24.9, *n* (%)	36 (37)	14 (31)	22 (42)
Overweight, 25.0–29.9, *n* (%)	34 (35)	17 (38)	17 (33)
Obese, ≥30.0, *n* (%)	24 (25)	14 (31)	10 (19)
**Body height (in.), mean (SD)**	168.35 ± 9.20	173.73 ± 65	162.84 ± 7.44

### 2.2 Image acquisition

Chest CT and PET‐CT scans were acquired using different protocols and scanners over a period of 10 years. The CT and PET‐CT scanners were primarily manufactured by GE Medical Systems and Siemens. The models of the CT scanners include Optima‐CT660, LightSpeed VCT, LightSpeed‐Ultra, Emotion, and Emotion‐Duo. The models of the PET‐CT scanners included Discovery ST, Emotion, and Emotion‐Duo. PET‐CT scans typically encompassed the body regions from the head to the mid‐thigh with a matrix size of 128 × 128 and an in‐plane resolution ranging from 4.1 to 5.5 mm. For the involved CT scans, the tube voltage was consistently 120 kV, the X‐ray tube current ranged from 40 to 498 mA, and the reconstruction kernels included “lung,” “soft,” and “standard” kernels. The image thickness of the chest CT scans acquired along with PET scans ranged from 2.5 to 4.0 mm.

### 2.3 Quantifying 3‐D body composition on the chest CT scans and whole‐body PET‐CT scans

**FIGURE 1:**
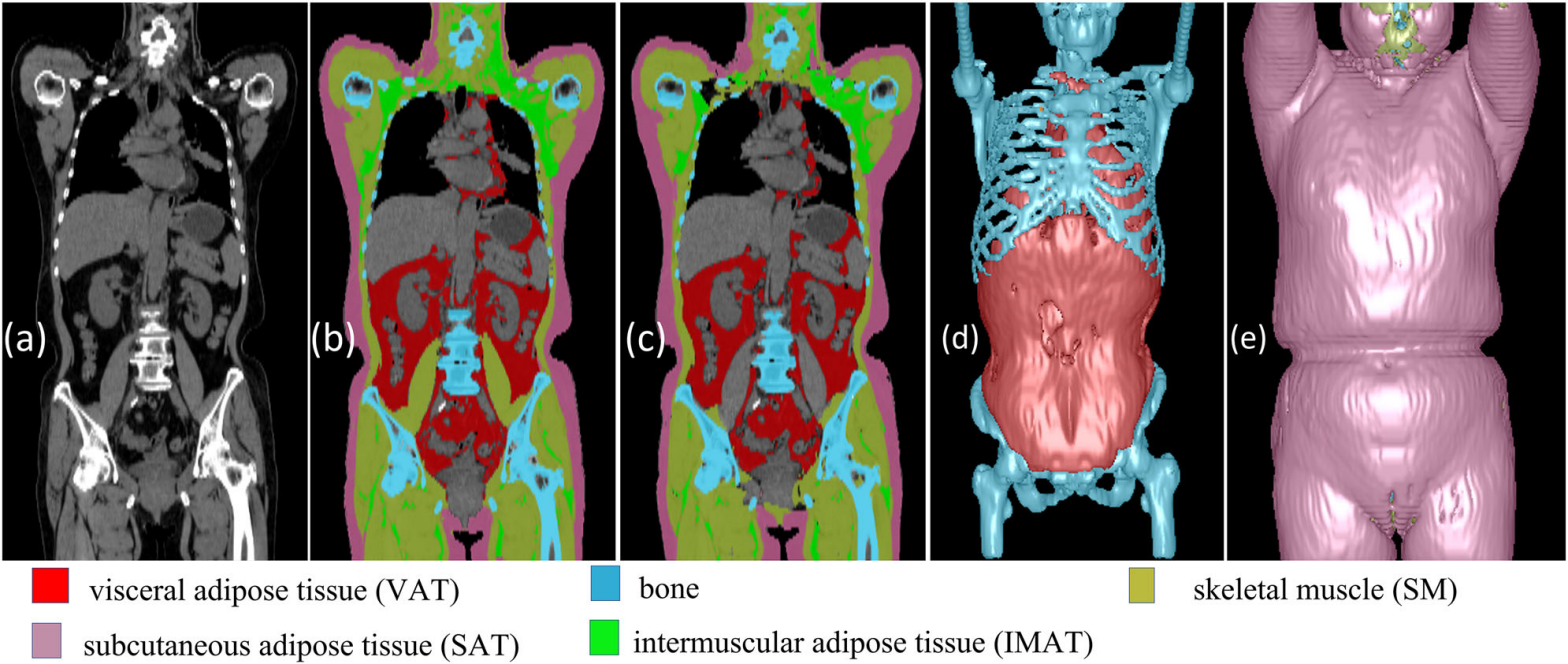
Computed results of body tissues on a whole‐body positron emission tomography‐computed tomography (PET‐CT) scan: (a) the original CT image, (b) the manual annotations of the body tissues, and (c) the computerized/automated segmentations of the body tissues. (d) and (e) The three‐dimensional (3‐D) visualization of the five body tissues

**FIGURE 2:**
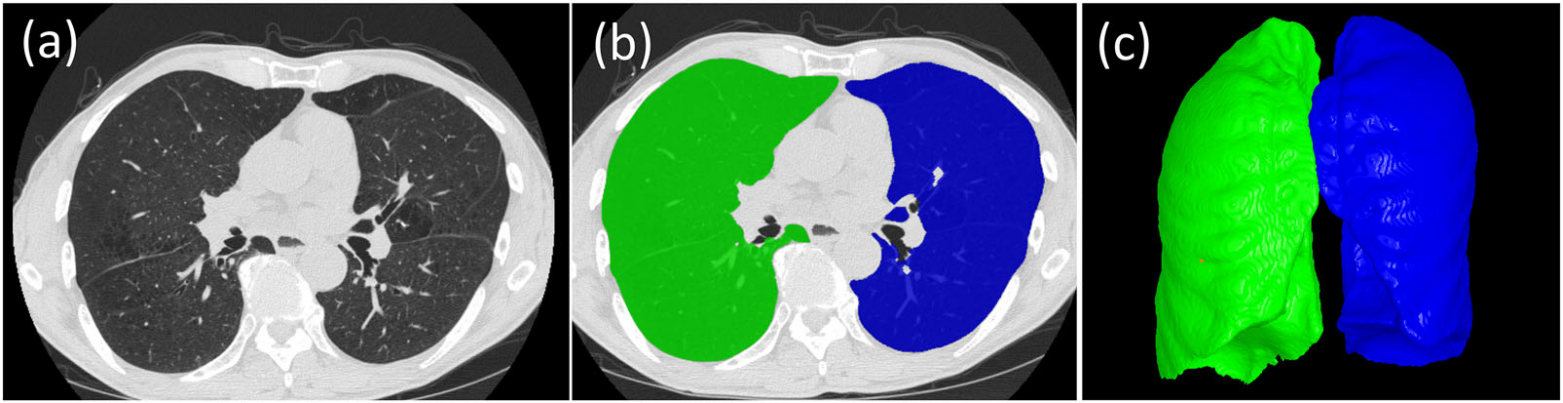
The lung volume segmentation: (a) original computed tomography (CT) images, (b) the segmented right and left lung regions in overlay, and (c) the three‐dimensional (3‐D) visualization of the segmented lungs

We developed a convolutional neural network (CNN)‐based algorithm to automatically segment five different tissues related to body composition depicted on CT images (VAT, SAT, IMAT, SM, and bone; Figure 1).^15^ This algorithm was trained and validated on a dataset consisting of 100 CT scans with manual annotations of these body tissues. The dataset included 50 whole‐body PET‐CT scans, 25 chest CT scans, and 25 abdominal CT scans, which were acquired on different subjects using various protocols. A 3‐D image patch‐based strategy was used to train the classical UNet model.^16^ The trained CNN model has been integrated into our in‐house software to automate the identification of the aforementioned five body tissues. As a standalone system, our in‐house software enables automated segmentation of several lung anatomical structures (e.g., lung regions, airways, and vessels) and the five body tissues. The performance of the CNN‐based algorithm for segmenting the five body tissues can be found in Ref. [15] and Tables S15 and S16. We used this in‐house software to segment the five body tissues (Figure 1) and lung volumes (Figure 2) depicted on the CT scans in the cohort. As a way to standardize the field‐of‐views of the CT scans, we quantified the volume and mean density (Hounsfield value) of the five body tissues located from vertebra T1 to L1 for the chest CT scans and those from vertebra C1 to the bottom of the pelvis for the whole‐body CT scans. The tissue volume and mean density measures were tabulated with subject demographics for prediction modeling. Figure 2 shows the results of the segmented body composition and lung regions. For comparison, we also quantified body tissues from a single CT slice at L3 and used these 2‐D measurements to estimate the 3‐D whole‐body volumes.

### 2.4 Prediction modeling

We used the stepwise multiple linear regression to develop the computer models for estimating whole‐body composition from chest CT scans or single CT images at L3 (from the PET‐CT scans) along with patient information. In addition to estimating the five body tissues, we also developed a model for estimating the total fat. There were three types of predictor variables: (1) the volumes and density of the five different body tissues computed from chest CT scans, (2) subject demographics (i.e., age, gender, weight, and height), and (3) lung volume computed from chest CT scans. For each whole‐body tissue measurement, the backward stepwise multiple linear regression started with a saturated model with all the predictor variables. In the regression analysis, variables with a *p*‐value less than 0.1 were included in the final prediction model. IBM SPSS v28 was used for the linear regression modeling.

### 2.5 Performance evaluation

We used the 10‐fold cross‐validation method to validate the performance of the prediction models. The data was split into 10‐folds. Ninefolds were used to develop the linear regression models. The remaining fold was used as an independent test set to evaluate the performance of the models. The training and testing processes were repeated 10 times to ensure that each case in the cohort was tested. Mean absolute differences (MADs), percentage errors, and the *R*‐squared (*R* ^2^) were computed as metrics to assess the prediction performance of the linear regression models. IBM SPSS v28 was used for the statistical analyses.

## 3 RESULTS

On chest CT scans, the computed average volumes and densities of SAT, VAT, IMAT, SM, and bone were summarized in Table S1. Independent‐samples *T*‐test showed statistically significant differences in the volume measures (*p* < 0.05) but no significant differences in the density measures of the body composition between male and female subjects based on either chest CT scans or PET‐CT scans. There were significant differences between the same tissue densities computed from chest CT scans and PET‐CT scans (*p* < 0.05). The scatter plots for the chest CT tissue volumes, the L3‐based tissue volumes, and their corresponding whole‐body CT tissue volumes were shown in Figures S1 and S2. We also provided the scatter plots to show the agreement between the body tissues measured on the chest CT scans and their corresponding regions on the whole‐body CT scans (Figure S3). The *R* ^2^ values ranged from 0.925 to 0.985 for the five body tissues.

The body composition areas computed from the single images at L3 (from PET‐CT scans) are summarized in Table S2. There were significant differences between the average density measures for VAT, IMAT, and SM computed from the entire chest CT scans versus the single images (*p* < 0.05). Although the images at L3 were obtained from PET‐CT scans, there were still significant differences between the density measurements obtained from L3 images and the entire PET‐CT scans. Like the measurements in Table S2, there were statistically significant differences in the areas (*p* < 0.05) but no significant differences in the densities of the body tissues between males and females as computed from the images at L3.

**FIGURE 3:**
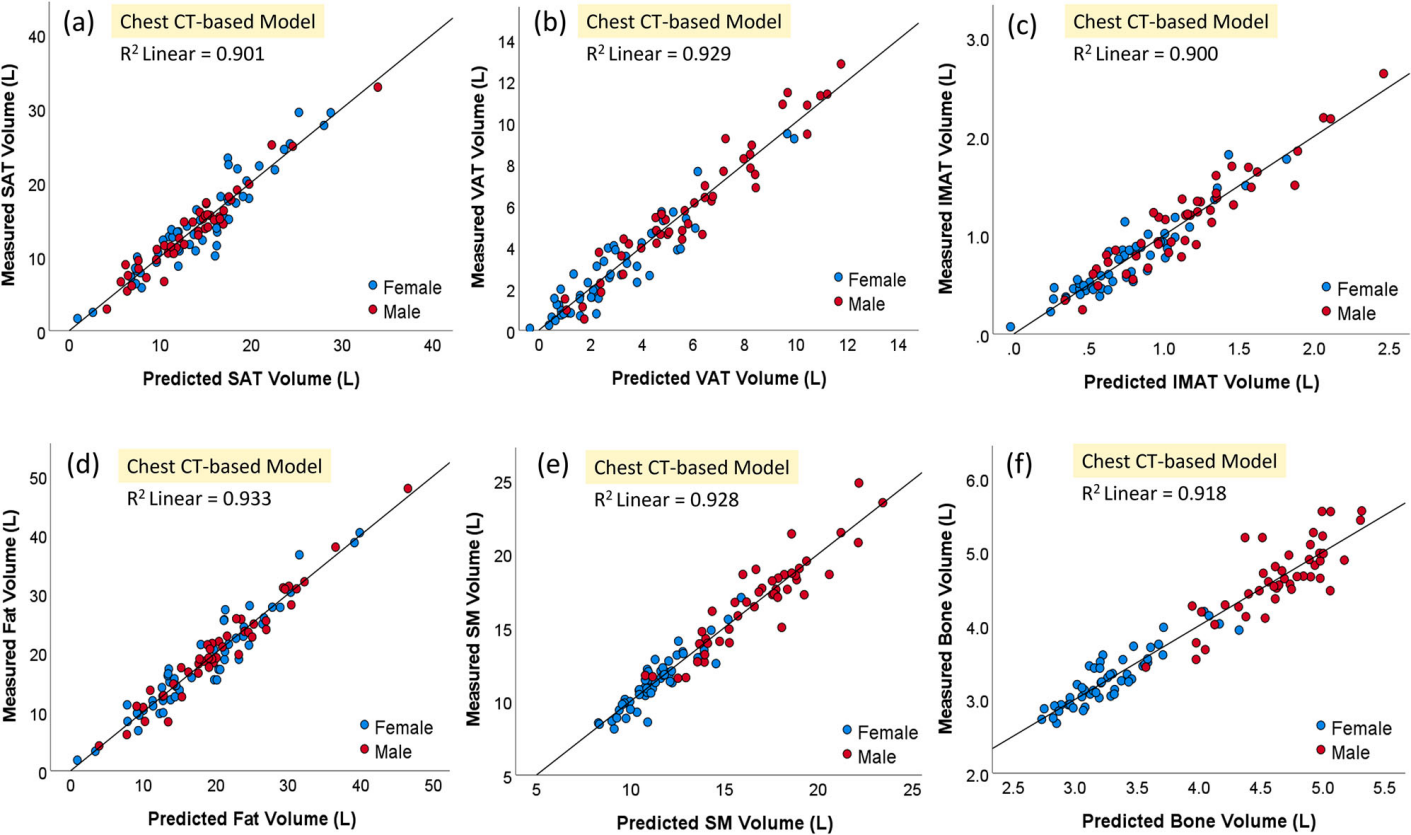
Scatter plots of the five body tissue volumes ((a): SAT, (b): VAT, (c): IMAT, (d): total fat, (e) SM, and (f) bone) computed from whole‐body positron emission tomography‐computed tomography (PET‐CT) scans versus estimated volumes from the chest CT‐based models. IMAT, intermuscular adipose tissue; SAT, subcutaneous adipose tissue; SM, skeletal muscle; VAT, visceral adipose tissue

**FIGURE 4:**
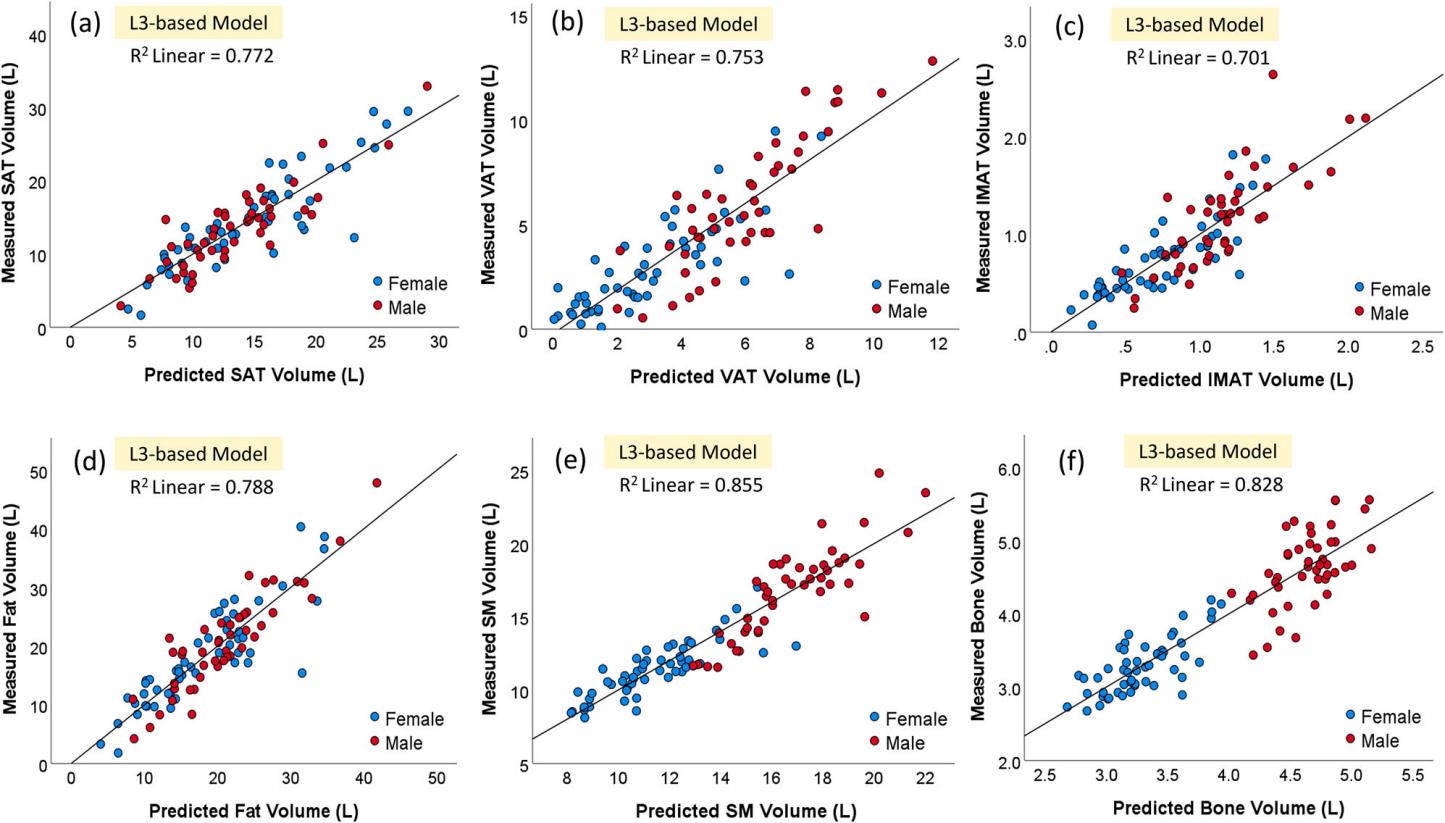
Scatter plots of the five body tissues ((a): SAT, (b): VAT, (c): IMAT, (d): total fat, (e) SM, and (f) bone) computed from whole‐body positron emission tomography‐computed tomography (PET‐CT) scans and body tissue areas predicted from the third lumbar (L3)‐based models. IMAT, intermuscular adipose tissue; SAT, subcutaneous adipose tissue; SM, skeletal muscle; VAT, visceral adipose tissue

**FIGURE 5:**
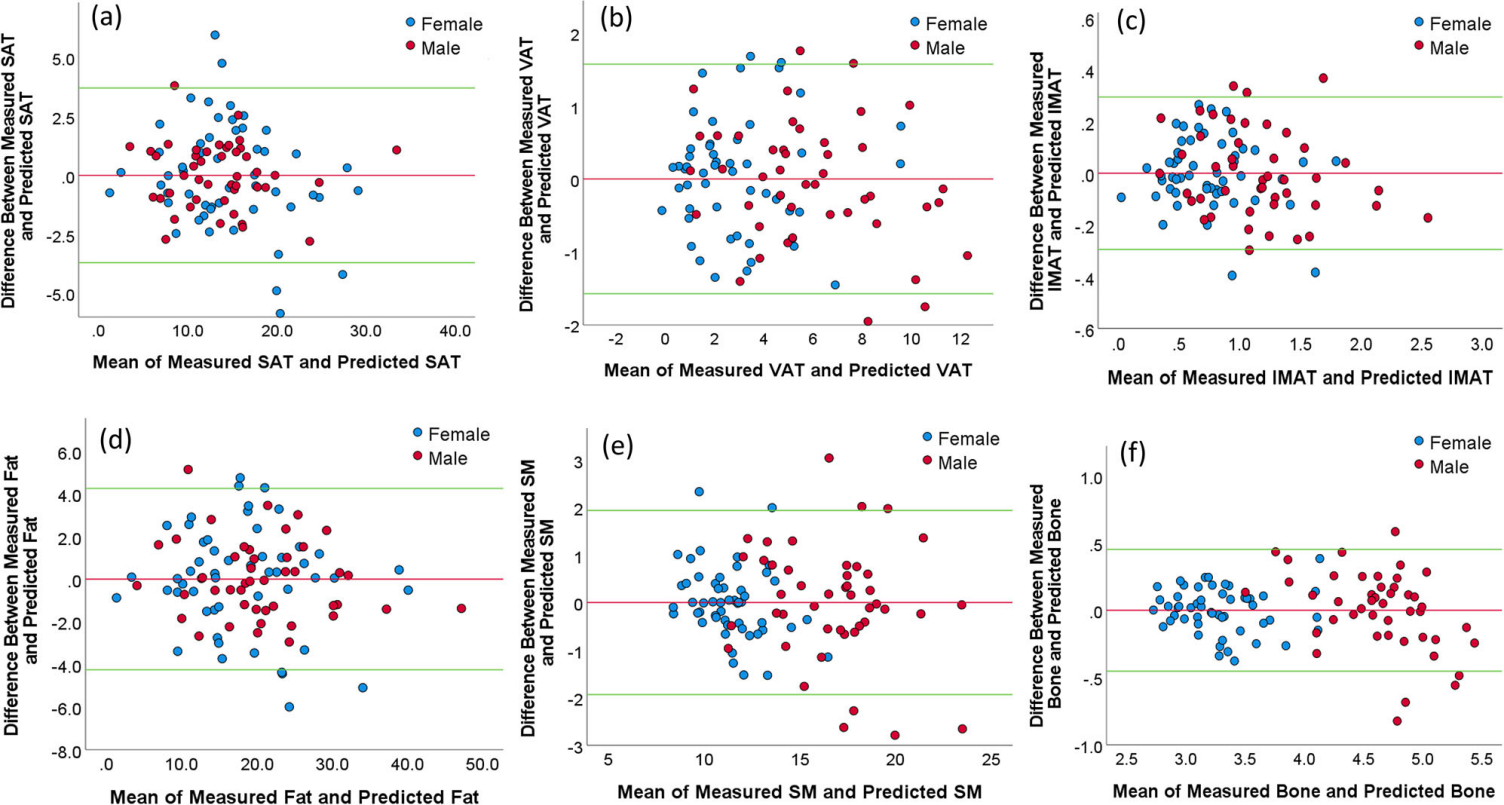
The Bland–Altman plots showing the agreement between the measured values (unit: L) from the whole‐body computed tomography (CT) scans and the predicted values from the chest CT‐based model for different body tissues ((a): SAT, (b): VAT, (c): IMAT, (d): total fat, (e) SM, and (f) bone). The horizontal red line shows the mean of the differences (=bias) between the two methods. The green horizontal lines show the upper and lower 95% limits of agreement (=bias ± 1.96 × SD).

**FIGURE 6:**
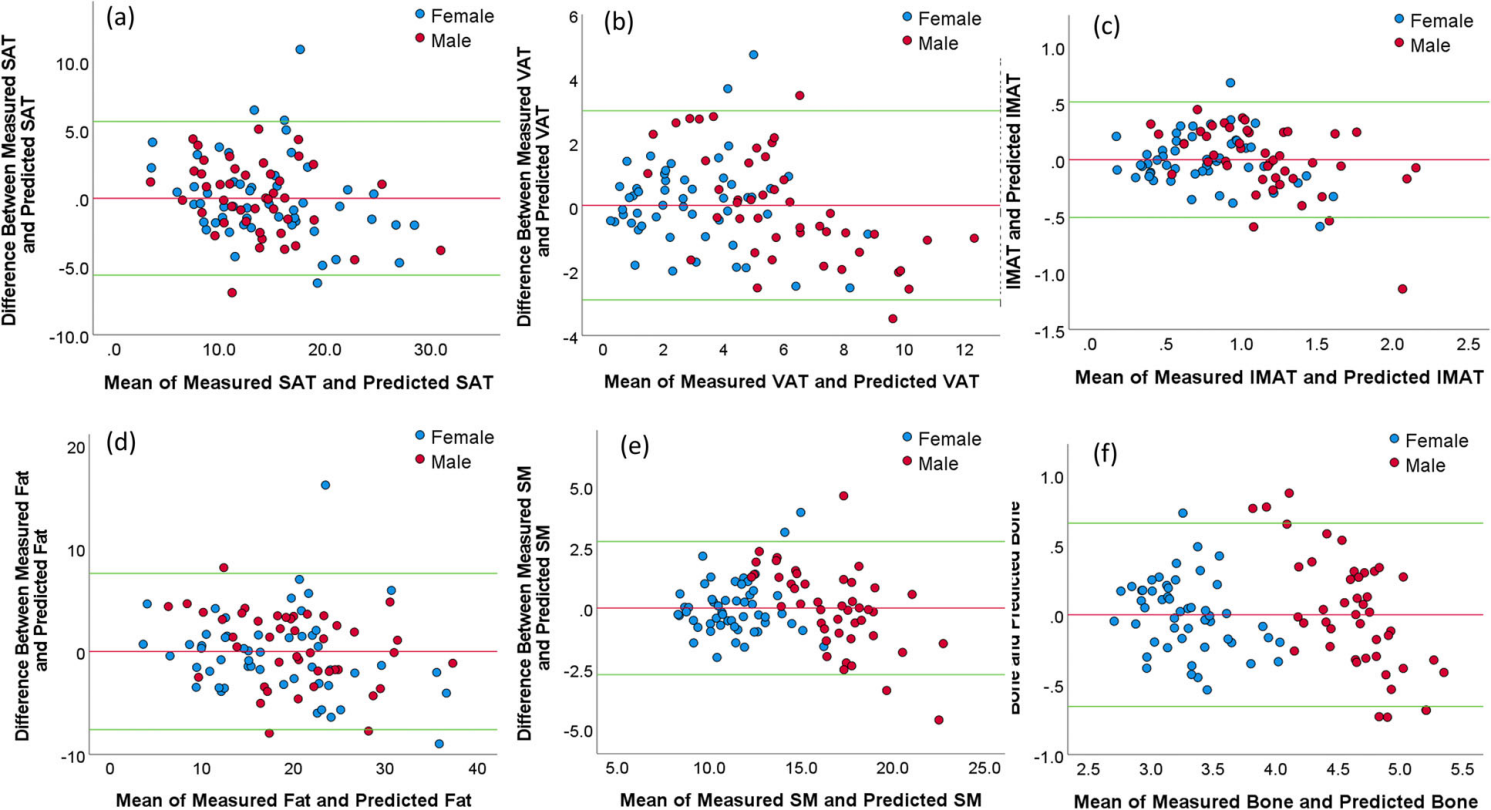
The Bland–Altman plots showing the agreement between the measured values (unit: L) from the whole‐body computed tomography (CT) scans and the predicted values (unit: L) from the third lumbar (L3)‐based model for different body ((a): SAT, (b): VAT, (c): IMAT, (d): total fat, (e) SM, and (f) bone). The horizontal red line shows the mean of the differences (=bias) between the two methods. The green horizontal lines show the upper and lower 95% limits of agreement (=bias ± 1.96 × SD).

The models based on chest CT scans demonstrated a significantly smaller MAD for estimating the volume of all body tissue measures based on whole‐body PET‐CT scans versus the models based on a single CT image at L3 (*p* < 0.05; Table 2). The chest CT‐based models also had a stronger linear correlation (0.901–0.933) for all variables with the whole‐body PET‐CT scan values versus the L3‐based models (0.701–0.855) (Figures 3 and 4). The corresponding residual plots were provided in Figures S3 and S4. Figures 5 and 6 showed the Bland–Altman plots of the agreement between the measured values (unit: L) from whole‐body CT scans and the predicted values (unit: L) from the chest CT‐based model and the L3‐based model, respectively. There was no bias between the volumes of the five tissue types computed from whole‐body PET‐CT scans and those estimated from the chest CT‐based models. The paired values of the two approaches fell essentially along the line of identity in the scatter plots. The linear equations for the chest CT scans and single CT image models are presented in Tables S3–S, ^14^.

**TABLE 2:** Performance of the computer models to estimate the volumes of the five whole‐body tissues from chest computed tomography (CT) scans and a single CT image at third lumbar (L3) in the positron emission tomography (PET)‐CT scans (*n* = 97)

	Chest CT scan			Single CT image at L3			
	MAD			MAD			
Tissue volume	L	%	*R*‐squared	L	%	*R*‐squared	*p*‐Value (*R*‐squared)
SAT	1.44 ± 1.21	12.21 ± 11.70	0.901	2.21 ± 1.83	22.62 ± 37.92	0.772	<0.001
VAT	0.63 ± 0.49	29.68 ± 61.99	0.929	1.17 ± 0.94	89.21 ± 276.31	0.753	<0.001
IMAT	0.12 ± 0.09	16.20 ± 18.42	0.900	0.20 ± 0.17	39.22 ± 41.78	0.701	<0.001
Total fat	1.65 ± 1.40	10.43 ± 10.79	0.933	3.01 ± 2.42	21.18 ± 37.46	0.788	<0.001
SM	0.71 ± 0.68	5.14 ± 4.75	0.928	1.03 ± 0.94	7.19 ± 6.43	0.855	<0.001
Bone	0.17 ± 0.15	4.32 ± 3.38	0.918	0.26 ± 0.21	6.20 ± 4.82	0.828	<0.001

*Note*: The manual annotations were used as the ground truth. The *p*‐value was computed to assess the performance difference based on chest CT scans and a single CT image at L3.

Abbreviations: CT, computed tomography; IMAT, intermuscular adipose tissue; L3, third lumbar; MAD, mean absolute difference; SAT, subcutaneous adipose tissue; SM, skeletal muscle; VAT, visceral adipose tissue; %, the percentage difference between the computerized results and the manual annotation.

Tables 3 and 4 show the prediction percentage errors when the subjects were classified into different categories in terms of BMI. For all BMI categories, the models based on chest CT scans demonstrated significantly smaller percentage errors for estimating all body tissue measures based on whole‐body PET‐CT scans than the models based on a single CT image at L3 (*p* < 0.05). For underweight subjects (*n* = 3), the prediction percentage error for VAT was extremely high but pretty low for SM and bone. The models demonstrated better performance in predicting the whole‐body fat volume, including SAT, VAT, and IMAT, for obese and overweight subjects than for the normal‐weight subjects.

**TABLE 3:** The percentage error (%) in estimating the volumes of the five whole‐body tissues from chest computed tomography (CT) scans (*n* = 97)

Body mass index	SAT	VAT	IMAT	SM	Bone
Underweight (BMI ≤ 18.5)	29.24 ± 21.37	197.88 ± 293.08	62.98 ± 62.84	4.14 ± 1.54	6.07 ± 3.70
Normal weight (BMI 18.5–24.9)	14.64 ± 12.02	39.31 ± 49.38	20.17 ± 17.68	4.98 ± 3.82	4.29 ± 3.08
Overweight (BMI 25.0–29.9)	11.48 ± 11.81	16.09 ± 11.21	12.33 ± 9.08	5.15 ± 5.39	4.34 ± 3.40
Obese (BMI ≥ 30.0)	8.35 ± 5.95	19.69 ± 23.41	11.28 ± 9.85	5.36 ± 4.87	4.05 ± 3.46

Abbreviations: BMI, body mass index; IMAT, intermuscular adipose tissue; SAT, subcutaneous adipose tissue; SM, skeletal muscle; VAT, visceral adipose tissue.

**TABLE 4:** The percentage error (%) in estimating the volumes of the five whole‐body tissues from a single computed tomography (CT) image at third lumbar (L3) in the positron emission tomography (PET)‐CT scans (*n* = 97)

Body mass index	SAT	VAT	IMAT	SM	Bone
Underweight (BMI ≤ 18.5)	116.85 ± 124.17	644.43 ± 972.06	115.62 ± 151.19	9.22 ± 7.54	5.98 ± 8.28
Normal weight (BMI 18.5–24.9)	21.84 ± 18.53	74.95 ± 90.14	28.24 ± 26.26	7.29 ± 5.27	6.35 ± 6.39
Overweight (BMI 25.0–29.9)	12.55 ± 11.96	23.12 ± 18.18	18.59 ± 14.91	6.57 ± 5.09	7.37 ± 4.93
Obese (BMI ≥ 30.0)	18.50 ± 19.60	32.18 ± 47.08	25.30 ± 24.64	8.90 ± 9.05	6.40 ± 3.91

Abbreviations: BMI, body mass index; IMAT, intermuscular adipose tissue; SAT, subcutaneous adipose tissue; SM, skeletal muscle; VAT, visceral adipose tissue.

## 4 DISCUSSION

As a major cause of morbidity and mortality, lung disease is extremely common in the United States and worldwide and carries a very high economic burden.^17^, ^18^ Lung disease is typically a lifelong condition that significantly affects a person's quality of life, and importantly, the incidence of lung disease will continue to rise over the next 50 years.^17^ In clinical practice, chest CT scans have been widely used for studying abnormal lung conditions. There have been numerous studies investigating the association between body composition depicted on CT scans and lung diseases (e.g., chronic obstructive pulmonary disease,^19^, ^20^, ^21^ COVID‐19,^22^ pulmonary fibrosis,^23^ and lung cancer^24^, ^25^). Many of these studies used multifrequency bioelectrical impedance analysis^19^, ^21^, ^22^ to assess body composition. Advances in artificial intelligence (AI) make it possible to efficiently and accurately quantify body composition depicted in a CT scan. However, most CT scans focus only on a local region of the body. Although we can directly quantify body composition based on chest CT scans, it is often desirable to know whole‐body composition, or at least that the regional body composition measures can accurately reflect whole‐body composition. In this way, the body composition can be assessed based on the availability of the chest CT scans without performing additional tests (e.g., DXA). To our knowledge, this is the first study that attempts to develop computer models for predicting five different 3‐D whole‐body composition volumes from chest CT scans. Given the prevalence of lung disease and its association with body composition, we believe that these computer models have important clinical utility.

The methods we used have several advantages over previous studies.^12^, ^13^, ^26^, ^27^, ^28^ First, we used a diverse dataset with paired chest CT scans and whole‐body PET‐CT scans. The CT scans were acquired using different protocols (e.g., scanner and dosage) over more than 10 years. Second, we quantified five different tissues related to body composition based on both chest and whole‐body PET‐CT scans using our AI algorithm. Most current studies related to body composition only quantified 1–3 tissue types due to the time‐consuming manual work that is required.^27^, ^28^, ^29^, ^30^, ^31^, ^32^, ^33^, ^34^ Ma et al.^26^ described a method to identify four different types of body tissues (i.e., SM, SAT, VAT, and bone), but the details about the dataset and the segmentation methods were missing. Third, we developed computer models to estimate whole‐body tissues from chest CT scans. For chest CT scans in our cohort, both the 3‐D measurements of body composition volumes and their 2‐D areas at L3 were computed to develop the prediction models separately. This comparison clarified the merits of using 3‐D chest CT measures for predicting whole‐body composition as compared with the traditional methods based on the single images at L3 (Table 2). Finally, the prediction models were developed based on the automated segmentation and quantification of body composition depicted on CT images. The relatively high prediction accuracy of the models reflects the reliability of our computer algorithms for quantifying body composition. We tested the contribution of lung volume to the model's performance by including it as a predictor variable. Our linear regression analyses showed that lung volume contributed to the estimation of the whole‐body SAT (*p* = 0.044) and bone (*p* = 0.039) but not to other prediction models (Tables S3 and S8).

The models based on body composition areas at L3 demonstrated a lower performance for estimating all five body tissues compared to the chest CT scan models (Table 2). L3‐based models demonstrated the best performance for estimating SM and bone compared to other body tissues (i.e., VAT, SAT, and IMAT). Age and/or gender contributed significantly to the estimation of SM and bones from the chest CT‐based models but not to adipose tissue when using predictor variables from the chest CT scans (Tables S7 and S8). When the body composition areas at L3 are used to predict the whole‐body composition, age and gender should not be ignored due to their significant contributions to the model (Tables S9–S14). We noticed that the computer models demonstrated very poor performance in predicting the whole‐body VAT and IMAT for the underweight subjects (Tables 3 and 4). First, there were only three subjects who were classified as underweight based on BMI. Second, for underweight subjects, the amount of VAT and IMAT could be very small in the body. These factors make it difficult to predict their whole‐body measures based on local body regions. In contrast, the percentage errors for predicting the SM and bone are pretty small and insensitive to BMI due to their relatively high amount in the body.

There are several limitations. We used a relatively small dataset to develop the computer models. Although only 97 paired scans were used, they were from the same subjects within 2 weeks of each other, ensuring no significant changes in body composition and thereby increasing reliability in analyses and prediction modeling. The dataset was from lung cancer patients, and it is not clear how this might affect the estimation of whole‐body compositions from chest CT scans. Our cohort was mostly white (89%; Table 1), and therefore our results may not be generalizable. In a strict sense, PET‐CT scans only cover the body regions from neck to thigh. Additionally, the CT portion in the PET‐CT examinations has lower image quality or resolution compared to regular CT scans. This image quality definitely affects the accuracy of the “ground truth.” However, the effect should be limited given the relatively large dimension of the body tissues (Figure S3). Although DXA scans cover the entire body, it is 2‐D and cannot provide a detailed volumetric quantification of body composition scans. As compared to DXA, the high resolution of CT imaging enables the identification of the fat infiltration in SM. Also, CT can distinguish visceral from subcutaneous fat with a higher level of precision.^35^, ^36^

## 5 CONCLUSIONS

We developed and validated a set of computer models to estimate five different whole‐body composition volumes from chest CT scans alone. Paired chest CT and whole‐body PET‐CT scans were used to develop and test the models. Our results demonstrated that whole‐body composition volumes can be reliably estimated from chest CT scans alone and were more accurate than methods that use a single CT slice.

## CONFLICT OF INTEREST

The authors declare that there is no conflict of interest that could be perceived as prejudicing the impartiality of the research reported.

## Supporting information

Figure S1 The scatter plots for the raw chest CT tissue volumes and the corresponding whole‐body CT tissue volumes

Figure S2 The scatter plots for the L3‐based body tissue areas and the corresponding whole‐body CT tissue volumes

Figure S3 The scatter plots for the quantified body tissues on the standardized chest CT scans and the corresponding PET‐CT‐derived “chest CT” regions

Figure S4 The residual plots for the predicted whole‐body tissue volumes (unit: L) from the chest CT‐based models

Figure S5 The residual plots for the predicted whole‐body tissue volumes (unit: L) from the L3‐based models

Table S1 Summary of the average volume and density measures of the five body tissues computed based on chest CT scans and whole‐body PET‐CT scans in our cohort
Table S2 Summary of the average area and density measures of the five body tissues computed from the image slices at L3 in the PET‐CT scans
Table S3 Linear regression model to estimate whole‐body SAT volume from a chest CT scan and subject demographics
Table S4 Linear regression model for predicting whole‐body VAT volume from a chest CT scan and patient information
Table S5 Linear regression model to estimate whole‐body IMAT volume from a chest CT scan and subject demographics
Table S6 Linear regression model to estimate total whole‐body fat volume from a chest CT scan and subject demographics
Table S7 Linear regression model to estimate total whole‐body muscle volume from a chest CT scan and subject demographics
Table S8 Linear regression model to estimate total whole‐body bone volume from a chest CT scan and subject demographics
Table S9 Linear regression model to estimate total whole‐body SAT volume from the image slice at L3 and subject demographics
Table S10 Linear regression model to estimate total whole‐body VAT volume from the image slice at L3 and subject demographics
Table S11 Linear regression model to estimate total whole‐body IMAT volume from the image slice at L3 and subject demographics
Table S12 Linear regression model to predict total whole‐body fat volume from the image slice at L3 and subject demographics
Table S13 Linear regression model to estimate total whole‐body muscle volume from the image slice at L3 and subject demographics
Table S14 Linear regression model to estimate total whole‐body bone volume from the image slice at L3 and subject demographics
Table S15 Summary of the performance of three CNN models in simultaneously segmenting five different body tissues separately in our cohort using the 10‐fold cross‐validation method
Table S16 The agreement between the computer results and the manual results of the five body tissues at L3

## Data Availability

Data is available from the corresponding author (RD) upon request.
