# Supplementary figures and images for: Estimating 3‐D whole‐body composition from a chest CT scan
Source: Med Phys. 2022 Jul 11;49(11):7108–17. doi: 10.1002/mp.15821 (PMC10084085; doi:10.1002/mp.15821)

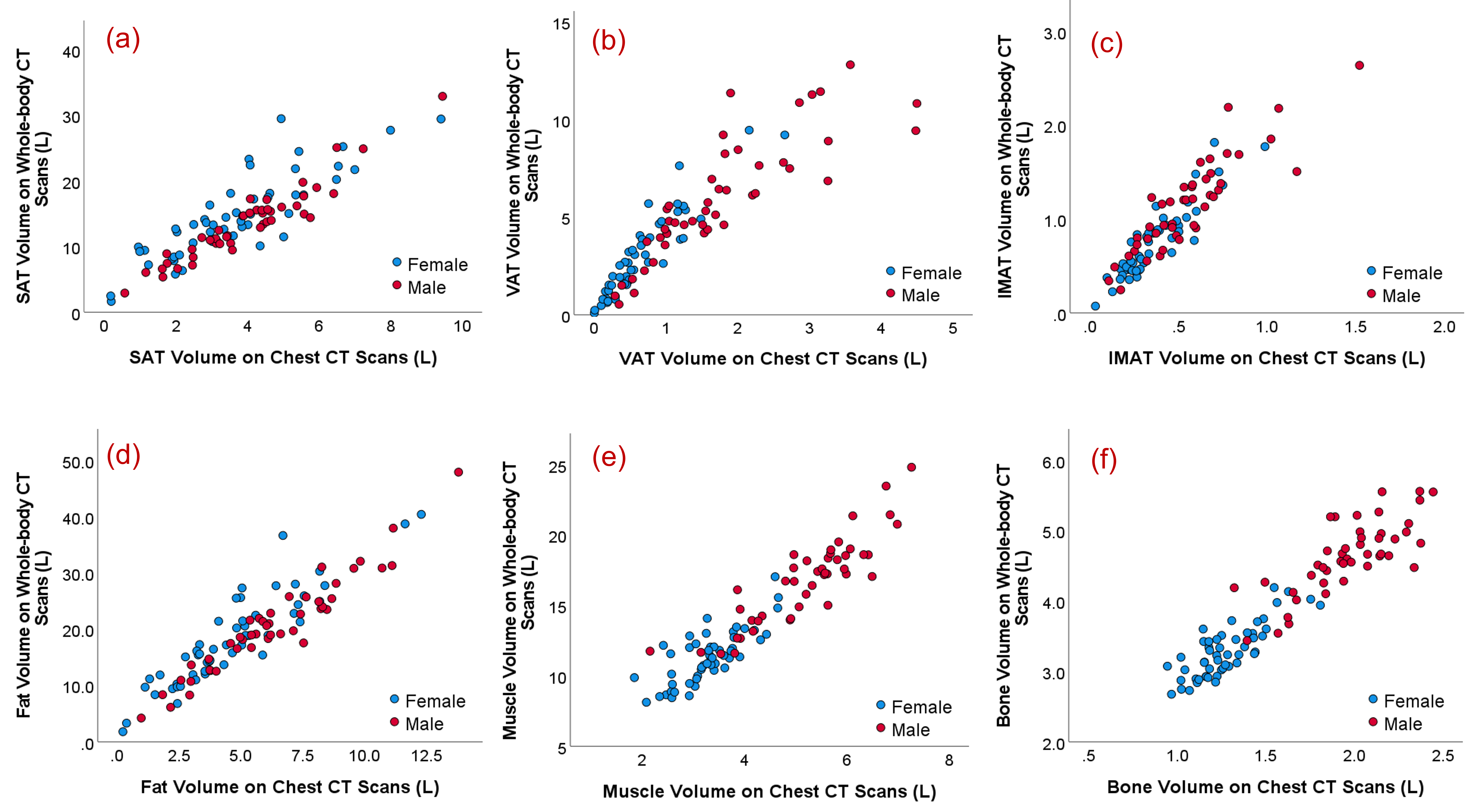

Supplement: Supplementary file 1 — Figure S1 The scatter plots for the raw chest CT tissue volumes and the corresponding whole‐body CT tissue volumes [file MP-49-7108-s002.tif]

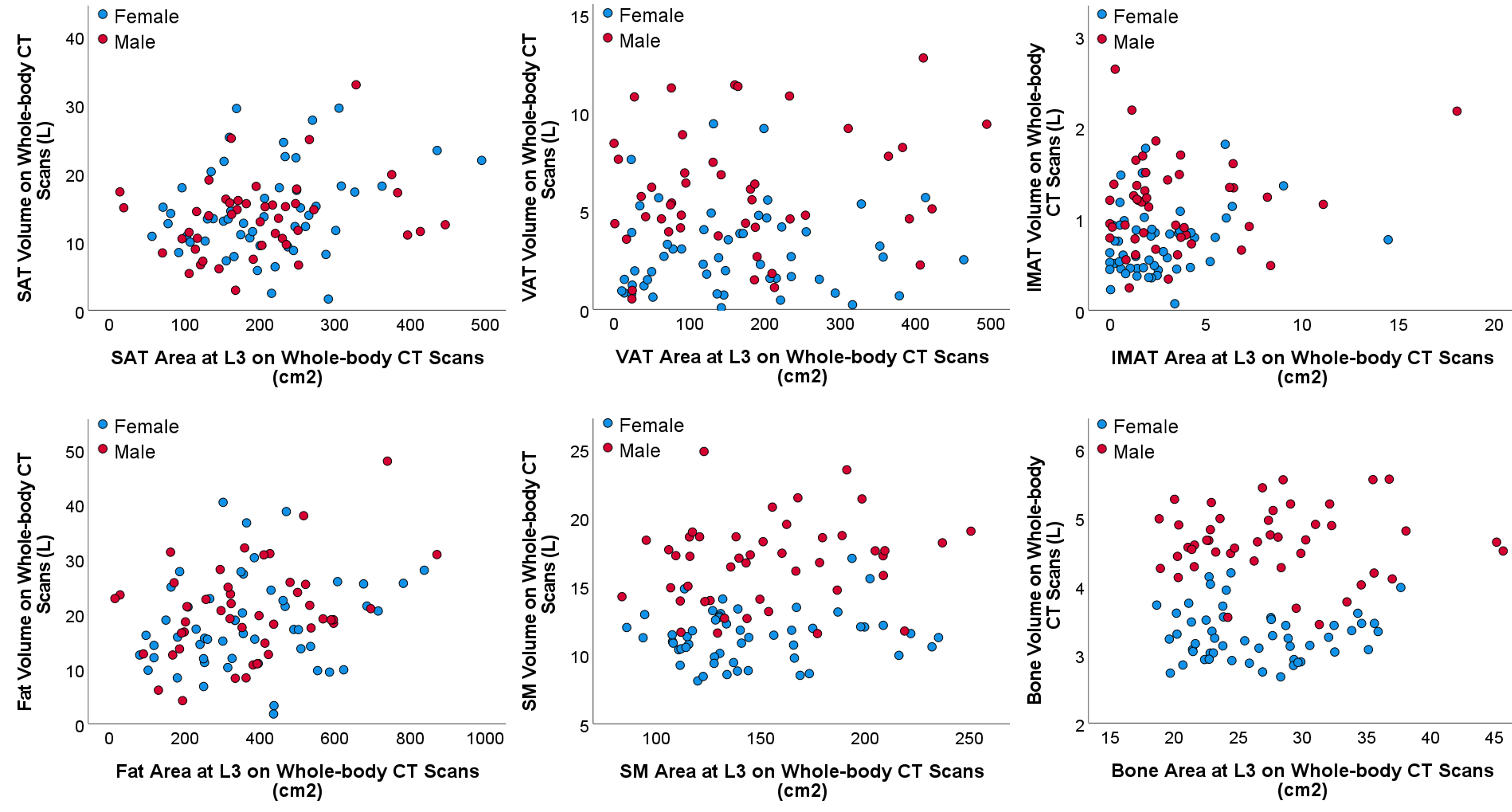

Supplement: Supplementary file 2 — Figure S2 The scatter plots for the L3‐based body tissue areas and the corresponding whole‐body CT tissue volumes [file MP-49-7108-s003.tif]

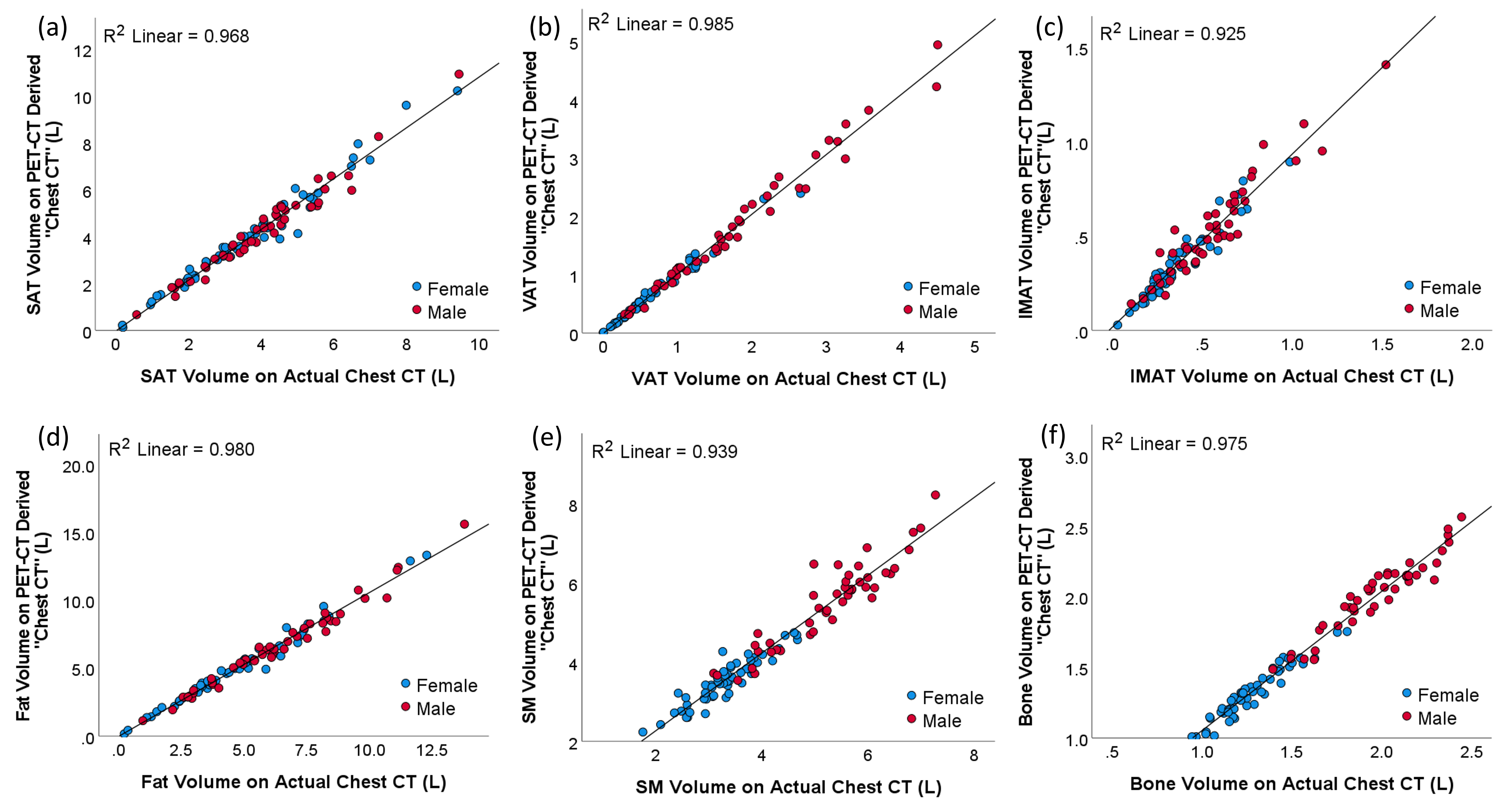

Supplement: Supplementary file 3 — Figure S3 The scatter plots for the quantified body tissues on the standardized chest CT scans and the corresponding PET‐CT‐derived “chest CT” regions [file MP-49-7108-s005.tif]

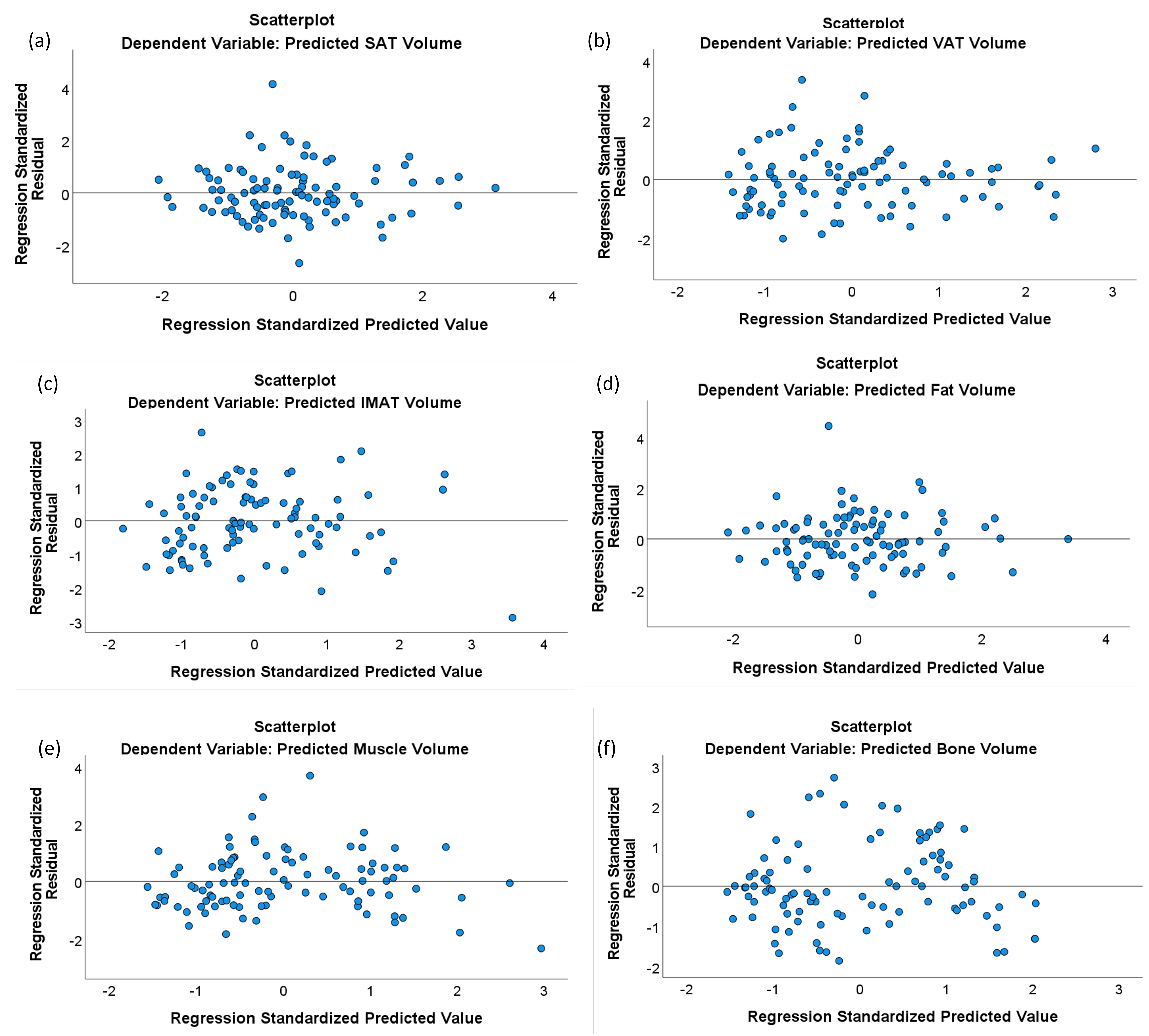

Supplement: Supplementary file 5 — Figure S5 The residual plots for the predicted whole‐body tissue volumes (unit: L) from the L3‐based models [file MP-49-7108-s001.tif]
