# Supplementary material for: Estimating 3‐D whole‐body composition from a chest CT scan
Source: Med Phys. 2022 Jul 11;49(11):7108–17. doi: 10.1002/mp.15821 (PMC10084085; doi:10.1002/mp.15821)
Supplement: Supplementary file 6 — Table S1 Summary of the average volume and density measures of the five body tissues computed based on chest CT scans and whole‐body PET‐CT scans in our cohort Table S2 Summary of the average area and density measures of the five body tissues computed from the image slices at L3 in the PET‐CT scans Table S3 Linear regression model to estimate whole‐body SAT volume from a chest CT scan and subject demographics Table S4 Linear regression model for predicting whole‐body VAT volume from a chest CT scan and patient information Table S5 Linear regression model to estimate whole‐body IMAT volume from a chest CT scan and subject demographics Table S6 Linear regression model to estimate total whole‐body fat volume from a chest CT scan and subject demographics Table S7 Linear regression model to estimate total whole‐body muscle volume from a chest CT scan and subject demographics Table S8 Linear regression model to estimate total whole‐body bone volume from a chest CT scan and subject demographics Table S9 Linear regression model to estimate total whole‐body SAT volume from the image slice at L3 and subject demographics Table S10 Linear regression model to estimate total whole‐body VAT volume from the image slice at L3 and subject demographics Table S11 Linear regression model to estimate total whole‐body IMAT volume from the image slice at L3 and subject demographics Table S12 Linear regression model to predict total whole‐body fat volume from the image slice at L3 and subject demographics Table S13 Linear regression model to estimate total whole‐body muscle volume from the image slice at L3 and subject demographics Table S14 Linear regression model to estimate total whole‐body bone volume from the image slice at L3 and subject demographics Table S15 Summary of the performance of three CNN models in simultaneously segmenting five different body tissues separately in our cohort using the 10‐fold cross‐validation method Table S16 The agreement between the computer results and [file MP-49-7108-s004.docx]

**Table S1**. Summary of the average volume and density measures of the five body tissues computed based on chest CT scans and whole-body PET-CT scans in our cohort. (Volume: Liter, Density: Hounsfield unit (HU))

| **Body tissue** | **Chest CT scans** | | | **Whole-body PET-CT scans** | | |
| --- | --- | --- | --- | --- | --- | --- |
|  | All  (n=97) | Male (n=45) | Female (n=52) | All  (n=97) | Male (n=45) | Female (n=52) |
| SAT volume | 3.8±1.8 | 4.0±1.7 | 3.7±1.9 | 14.1±6.0 | 13.5±5.6 | 14.5±6.4 |
| SAT density | -93.2±11.2 | -91.3±11.2 | -94.8±12.9 | -94.6±9.1 | -92.3±8.1 | -96.6±9.6 |
| VAT volume | 1.2±1.0 | 1.8±1.0 | 0.6±0.5 | 4.3±3.0 | 6.0±3.0 | 2.9±2.2 |
| VAT density | -92.7±6.5 | -94.6±5.9 | -91.0±6.7 | -89.6±7.3 | -90.7±6.6 | -88.7±7.8 |
| IMAT volume | 0.4±0.3 | 0.6±0.3 | 0.4±0.2 | 0.9±0.5 | 1.2±0.5 | 0.7±0.4 |
| IMAT density | -90.6±7.4 | -89.1±7.8 | -91.9±6.9 | -81.9±11.4 | -82.8±6.7 | -81.2±14.3 |
| SM volume | 4.2±1.3 | 5.2±1.1 | 3.3±0.6 | 13.93.7 | 16.8±3.1 | 11.4±1.9 |
| SM density | 23.0±8.4 | 24.7±6.7 | 21.6±9.5 | 33.9±9.5 | 34.5±9.1 | 33.4±9.8 |
| Bone volume | 1.6±0.4 | 2.0±0.3 | 1.3±0.2 | 3.9±0.8 | 4.6±0.5 | 3.3±0.4 |
| Bone density | 287.4±47.7 | 284.3±44.7 | 290.1±50.4 | 333.8±65.4 | 328.9±43.1 | 338.0±80.0 |

SAT – subcutaneous adipose tissue, VAT – visceral adipose tissue, IMAT – intermuscular adipose tissue, SM – skeletal muscle

**Table S2**. Summary of the average area and density measures of the five body tissues computed from the image slices at L3 in the PET-CT scans. (Area in cm^2^, Density in Hounsfield unit (HU))

| **Body tissue** | **Measures at L3** | | |
| --- | --- | --- | --- |
|  | All (n=97) | Male (n=45) | Female (n=52) |
|  |  |  |  |
| SAT area | 204.5±92.7 | 200±97.4 | 208.5±89.2 |
| SAT density | -97.3±10.7 | -96.9±12.3 | -97.7±9.3 |
| VAT area | 162.2±122.2 | 164.5±131.1 | 160.2±115.2 |
| VAT density | -86.9±13.1 | -84.9±16.9 | -88.6±8.5 |
| IMAT area | 2.9±3.0 | 3.3±3.4 | 2.5±2.6 |
| IMAT density | -69.7±20.4 | -69.8±21.1 | -69.6±20.0 |
| SM area | 149.5±38.7 | 153.3±39.9 | 146.2±37.7 |
| SM density | 30.8±11.0 | 32±12.0 | 29.7±10.1 |
| Bone area | 27.0±5.8 | 27.8±6.6 | 26.4±5.0 |
| Bone density | 274.5±58.9 | 281±61.9 | 268.8±56.1 |

SAT – subcutaneous adipose tissue, VAT – visceral adipose tissue, IMAT – intermuscular adipose tissue, SM – skeletal muscle

**Table S3:** Linear regression model to estimate whole-body SAT volume from a chest CT scan and subject demographics.

| **Model** | **Beta coefficient (B)** | **p-value** | **95% CI for B** |
| --- | --- | --- | --- |
| (Constant) | -28.436 | <0.001 | (-43.842,-13.030) |
| BMI (kg/m2) | 0.799 | <0.001 | 0.659,0.940) |
| Height (cm) | 0.121 | 0.002 | (0.045,0.196) |
| Lung volume | -0.538 | 0.044 | (-1.066,-0.009) |
| SAT volume | 1.755 | <0.001 | (1.341,2.168) |
| Muscle volume | -1.432 | <0.001 | (-1.972,-0.892) |
| Bone density | -0.011 | 0.091 | (-0.023,0.002) |
| IMAT volume | -0.109 | 0.076 | (-0.229,0.011) |
| VAT volume/total volume | -19.2 | 0.003 | (-31.728,-6.671) |

SAT – subcutaneous adipose tissue, VAT – visceral adipose tissue, IMAT – intermuscular adipose tissue, SM – skeletal muscle

**Table S4.** The linear regression model for predicting whole-body VAT volume from a chest CT scan and patient information.

| **Model** | **Beta coefficient (B)** | **p-value** | **95% CI for B** |
| --- | --- | --- | --- |
| (Constant) | 9.209 | 0.038 | (0.540,17.879) |
| Height | 0.046 | 0.001 | (0.019,0.073) |
| BMI | 0.06 | 0.033 | (0.005,0.114) |
| VAT volume | 1.03 | <.001 | (0.712,1.348) |
| VAT density | -0.114 | <.001 | (-0.165,-0.063) |
| SAT density | 0.033 | 0.01 | (0.008,0.058) |
| IMAT volume | 5.153 | <.001 | (3.883,6.422) |
| IMAT density | 0.08 | 0.001 | (0.032,0.127) |
| Bone volume | -2.779 | <.001 | (-3.839,-1.719) |
| Muscle volume/total volume | -22.618 | <.001 | (-31.981,-13.255) |
| Total volume | 0.197 | 0.01 | (0.049,0.346) |

SAT – subcutaneous adipose tissue, VAT – visceral adipose tissue, IMAT – intermuscular adipose tissue, SM – skeletal muscle

**Table S5.** Linear regression model to estimate whole-body IMAT volume from a chest CT scan and subject demographics.

| **Model** | **Beta coefficient (B)** | **p-value** | **95% CI for B** |
| --- | --- | --- | --- |
| (Constant) | 1.121 | 0.126 | (-0.319,2.562) |
| Height | 0.008 | 0.002 | (0.003,0.013) |
| IMAT volume | 1.863 | <.001 | (1.635,2.092) |
| IMAT density | 0.016 | <.001 | (0.008,0.024) |
| Muscle volume | -0.069 | <.001 | (-0.099,-0.039) |
| Muscle density | -0.008 | 0.008 | (-0.014,-0.002) |
| Muscle volume/total volume | -1.169 | 0.105 | (-2.588,0.250) |
| BMI | 0.014 | 0.005 | (0.004,0.024) |

SAT – subcutaneous adipose tissue, VAT – visceral adipose tissue, IMAT – intermuscular adipose tissue, SM – skeletal muscle

**Table S6.** Linear regression model to estimate total whole-body fat volume from a chest CT scan and subject demographics.

| **Model** | **Beta coefficient (B)** | **p-value** | **95% CI for B** |
| --- | --- | --- | --- |
| (Constant) | -49.274 | <.001 | (-68.766,-29.781) |
| Height | 0.176 | <.001 | (0.091,0.261) |
| BMI | 0.851 | <.001 | (0.689,1.013) |
| Lung volume | -0.419 | 0.147 | (-0.989,0.151) |
| VAT density | -0.1 | 0.162 | (-0.240,0.041) |
| Muscle volume | -2.009 | <.001 | (-2.613,-1.405) |
| Total volume | 1.795 | <.001 | (1.391,2.198) |
| SAT volume/total volume | 9.893 | 0.003 | (3.407,16.379) |
| IMAT volume/total volume | 64.07 | 0.036 | (4.287,123.852) |

SAT – subcutaneous adipose tissue, VAT – visceral adipose tissue, IMAT – intermuscular adipose tissue, SM – skeletal muscle

**Table S7.** Linear regression model to estimate total whole-body muscle volume from a chest CT scan and subject demographics.

| **Model** | **Beta coefficient (B)** | **p-value** | **95% CI for B** |
| --- | --- | --- | --- |
| (Constant) | 53.981 | 0.095 | (-9.592,117.555) |
| Sex | 4.487 | <.001 | (2.882,6.093) |
| Height | 0.17 | <.001 | (0.109,0.231) |
| BMI | 0.394 | <.001 | (0.276,0.512) |
| VAT density | -0.101 | 0.073 | (-0.212,0.010) |
| SAT density | 0.073 | 0.078 | (-0.008,0.154) |
| Bone volume | -4.645 | 0.048 | (-9.253,-0.037) |
| Total volume | 0.563 | 0.065 | (-0.035,1.161) |
| Muscle volume/total volume | -90.246 | 0.046 | (-178.873,-1.618) |
| VAT volume/total volume | -15.116 | 0.026 | (-28.421,-1.811) |

SAT – subcutaneous adipose tissue, VAT – visceral adipose tissue, IMAT – intermuscular adipose tissue, SM – skeletal muscle

**Table S8.** Linear regression model to estimate total whole-body bone volume from a chest CT scan and subject demographics.

| **Model** | **Beta coefficient (B)** | **p-value** | **95% CI for B** |
| --- | --- | --- | --- |
| (Constant) | -6.013 | <.001 | (-8.148,-3.877) |
| Sex | 0.584 | <.001 | (0.341,0.827) |
| Height | 0.039 | <.001 | (0.028,0.050) |
| BMI | 0.049 | <.001 | (0.028,0.069) |
| Age | 0.01 | 0.008 | (0.003,0.017) |
| Lung volume | 0.08 | 0.039 | (0.006,0.154) |
| SAT volume | -0.174 | <.001 | (-0.265,-0.084) |
| Bone volume | 0.654 | <.001 | (0.386,0.921) |
| Bone density | 0.001 | 0.13 | (0.000,0.003) |
| SAT volume/total volume | 2.003 | 0.02 | (0.328,3.679) |

SAT – subcutaneous adipose tissue, VAT – visceral adipose tissue, IMAT – intermuscular adipose tissue, SM – skeletal muscle

**Table S9.** Linear regression model to estimate total whole-body SAT volume from the image slice at L3 and subject demographics.

| **Model** | **Beta coefficient (B)** | **p-value** | **95% CI for B** |
| --- | --- | --- | --- |
| (Constant) | -41.956 | 0.278 | (-118.346,34.435) |
| Age | 0.06 | 0.072 | (-0.005,0.125) |
| Sex | -4.125 | <.001 | (-5.908,-2.341) |
| Height | 0.457 | 0.021 | (0.071,0.844) |
| Weight | -0.464 | 0.034 | (-0.892,-0.036) |
| BMI | 2.432 | <.001 | (1.219,3.645) |
| IMAT area | 0.259 | 0.078 | (-0.030,0.548) |
| Bone area | -0.263 | 0.007 | (-0.451,-0.074) |
| Muscle area/all tissue area | -68.47 | 0.005 | (-115.571,-21.370) |
| IMAT area/area fat area | -72.419 | 0.016 | (-130.831,-14.007) |
| Muscle area/all tissue area | 21.446 | 0.003 | (7.687,35.206) |
| All tissue area | 0.011 | 0.015 | (0.002,0.020) |
| VAT area/all tissue area | 5.319 | 0.145 | (-1.880,12.517) |

SAT – subcutaneous adipose tissue, VAT – visceral adipose tissue, IMAT – intermuscular adipose tissue, SM – skeletal muscle

**Table S10.** Linear regression model to estimate total whole-body VAT volume from the image slice at L3 and subject demographics.

| **Model** | **Beta coefficient (B)** | **p-value** | **95% CI for B** |
| --- | --- | --- | --- |
| (Constant) | 2.848 | 0.541 | (-6.369,12.065) |
| Age | 0.059 | <.001 | (0.025,0.094) |
| Sex | 1.842 | <.001 | (0.907,2.777) |
| Height | -0.075 | 0.006 | (-0.127,-0.022) |
| Weight | 0.13 | <.001 | (0.107,0.152) |
| Muscle density | -0.024 | 0.126 | (-0.055,0.007) |

SAT – subcutaneous adipose tissue, VAT – visceral adipose tissue, IMAT – intermuscular adipose tissue, SM – skeletal muscle

**Table S11.** Linear regression model to estimate total whole-body IMAT volume from the image slice at L3 and subject demographics.

| **Model** | **Beta coefficient (B)** | **p-value** | **95% CI for B** |
| --- | --- | --- | --- |
| (Constant) | 1.448 | 0.111 | (-0.339,3.234) |
| Age | 0.01 | 0.004 | (0.003,0.016) |
| Sex | 0.302 | 0.002 | (0.119,0.485) |
| Height | -0.016 | 0.003 | (-0.026,-0.005) |
| Weight | 0.021 | <.001 | (0.017,0.025) |
| Muscle density | -0.008 | 0.009 | (-0.015,-0.002) |
| IMAT/total fat area | -3.472 | 0.12 | (-7.862,0.918) |

SAT – subcutaneous adipose tissue, VAT – visceral adipose tissue, IMAT – intermuscular adipose tissue, SM – skeletal muscle

**Table S12.** Linear regression model to predicting total whole-body fat volume from the image slice at L3 and subject demographics.

| **Model** | **Beta coefficient (B)** | **p-value** | **95% CI for B** |
| --- | --- | --- | --- |
| (Constant) | -34.627 | 0.005 | (-58.794,-10.460) |
| Age | 0.125 | 0.003 | (0.044,0.206) |
| Sex | -1.9 | 0.096 | (-4.143,0.344) |
| BMI | 1.595 | <.001 | (1.439,1.750) |
| Height | 0.097 | 0.11 | (-0.022,0.216) |
| Muscle density | -0.149 | 0.005 | (0.252,-0.047) |
| IMAT area/total fat area | -67.412 | 0.059 | (-137.564,2.739) |

SAT – subcutaneous adipose tissue, VAT – visceral adipose tissue, IMAT – intermuscular adipose tissue, SM – skeletal muscle

**Table S13.** Linear regression model to estimate total whole-body muscle volume from the image slice at L3 and subject demographics.

| **Model** | **Beta coefficient (B)** | **p-value** | **95% CI for B** |
| --- | --- | --- | --- |
| (Constant) | 17.859 | 0.004 | (5.826,29.892) |
| Age | -0.051 | 0.006 | (-0.087,-0.015) |
| Sex | 4.53 | <.001 | (3.717,5.343) |
| Weight | 0.135 | <.001 | (0.112,0.158) |
| IMAT area | -0.121 | 0.061 | (-0.248,0.006) |
| Muscle area /total tissue area | -12.293 | 0.074 | (-25.811,1.225) |
| SAT area/VAT area | -0.171 | 0.055 | (-0.346,0.004) |

SAT – subcutaneous adipose tissue, VAT – visceral adipose tissue, IMAT – intermuscular adipose tissue, SM – skeletal muscle

**Table S14.** Linear regression model to estimate total whole-body bone volume from the image slice at L3 and subject demographics.

| **Model** | **Beta coefficient (B)** | **p-value** | **95% CI for B** |
| --- | --- | --- | --- |
| (Constant) | -4.923 | <.001 | (-7.113,-2.733) |
| Age | 0.006 | 0.101 | (-0.001,0.014) |
| Sex | 0.851 | <.001 | (0.648,1.055) |
| Weight | 0.007 | 0.004 | (0.002,0.012) |
| Height | 0.044 | <.001 | (0.033,0.056) |
| VAT area | 0.001 | 0.022 | (0.000,0.002) |
| VAT density | -0.006 | 0.135 | (-0.014,0.002) |
| Muscle area | -0.006 | 0.002 | (-0.009,-0.002) |
| Muscle density | 0.01 | 0.078 | (-0.001,0.020) |
| Bone area | 0.019 | 0.038 | (0.001,0.037) |
| IMAT area | -0.026 | 0.148 | (-0.061,0.009) |

SAT – subcutaneous adipose tissue, VAT – visceral adipose tissue, IMAT – intermuscular adipose tissue, SM – skeletal muscle

**Table S15**. Summary of the performance of three CNN models in simultaneously segmenting five different body tissues separately in our cohort using the 10-fold cross-validation method.

| **Method** | **Metrics** | **VAT** | **SAT** | **IMAT** | **SM** | **Bone** |
| --- | --- | --- | --- | --- | --- | --- |
| UNet | Dice coefficient | 0.819±0.099 | 0.896±0.082 | 0.590±0.089 | 0.871±0.030 | 0.870±0.036 |
|  | Jaccard index | 0.705±0.126 | 0.819±0.109 | 0.424±0.086 | 0.772±0.046 | 0.771±0.055 |
| R2Unet | Dice coefficient | 0.744±0.154 | 0.863±0.117 | 0.433±0.109 | 0.857±0.033 | 0.808±0.045 |
|  | Jaccard index | 0.613±0.175 | 0.773±0.140 | 0.282±0.089 | 0.750±0.050 | 0.680±0.061 |
| UNet++ | Dice coefficient | 0.749±0.142 | 0.856±0.123 | 0.469±0.103 | 0.838±0.035 | 0.803±0.052 |
|  | Jaccard index | 0.616±0.162 | 0.763±0.143 | 0.312±0.088 | 0.723±0.051 | 0.677±0.069 |

**Table S16**. The agreement between the computer results and the manual results of the five body tissues at L3.

| **Method** | **Metrics** | **VAT** | **SAT** | **IMAT** | **SM** | **Bone** |
| --- | --- | --- | --- | --- | --- | --- |
| UNet | Dice coefficient | 0.892±0.079 | 0.941±0.080 | 0.720±0.081 | 0.939±0.028 | 0.954±0.037 |
|  | Jaccard index | 0.805±0.099 | 0.851±0.099 | 0.641±0.075 | 0.852±0.040 | 0.871±0.046 |
|  | MAD | 5.110±6.132 | 6.181±9.566 | 1.671±1.508 | 6.913±7.917 | 3.828±1.729 |
| R2Unet | Dice coefficient | 0.849±0.144 | 0.922±0.097 | 0.573±0.100 | 0.930±0.030 | 0.927±0.044 |
|  | Jaccard index | 0.729±0.149 | 0.836±0.114 | 0.428±0.080 | 0.837±0.044 | 0.852±0.059 |
|  | MAD | 5.441±7.099 | 6.319±9.890 | 2.086±2.005 | 7.018±7.994 | 3.978±1.764 |
| UNet++ | Dice coefficient | 0.830±0.137 | 0.916±0.119 | 0.599±0.097 | 0.928±0.033 | 0.932±0.049 |
|  | Jaccard index | 0.727±0.150 | 0.825±0.116 | 0.465±0.080 | 0.862±0.044 | 0.867±0.056 |
|  | MAD | 5.766±7.135 | 6.322±9.992 | 1.992±1.744 | 7.011±7.995 | 3.887±1.731 |

SAT – subcutaneous adipose tissue, VAT – visceral adipose tissue, IMAT – intermuscular adipose tissue, SM – skeletal muscle, MAD: mean absolute difference (unit: cm^2^)
